# Infrared Thermography in the Architectural Field

**DOI:** 10.1155/2013/323948

**Published:** 2013-11-10

**Authors:** Carosena Meola

**Affiliations:** Department of Industrial Engineering, Aerospace Division, University of Naples Federico II, Via Claudio 21, 80125 Napoli, Italy

## Abstract

Infrared thermography is becoming ever more popular in civil engineering/architecture mainly due to its noncontact character which includes two great advantages. On one side, it prevents the object, under inspection, from any alteration and this is worthwhile especially in the presence of precious works of art. On the other side, the personnel operate in a remote manner far away from any hazard and this complies well with safety at work regulations. What is more, it offers the possibility to quickly inspect large surfaces such as the entire facade of a building. This paper would be an overview of the use of infrared thermography in the architectural and civil engineering field. First, some basic testing procedures are described, and then some key examples are presented owing to both laboratory tests and applications in situ spanning from civil habitations to works of art and archaeological sites.

## 1. Introduction 

Infrared thermography (IRT) is being used in an ever more broad number of application fields and for many different purposes; indeed, any process, which is temperature dependent, may benefit from the use of an infrared device. In other words, an infrared imaging device should be considered a precious ally to consult for diagnostics and preventative purposes, for the understanding of complex fluid dynamics phenomena, or for material characterization and procedures assessment which can help improve the design and fabrication of products. Infrared thermography may accompany the entire life of a product, since it may be used to control the manufacturing process, to nondestructively assess the final product integrity, and to monitor the component in-service. 

The first use of infrared thermography, as a nondestructive testing technique, dates back to the beginning of the last century [1, 2], but it was only recently accepted amongst standardized techniques. Initially, IRT suffered from perplexities and incomprehension mainly because of difficulties in the interpretation of thermograms. It received renewed attention starting from the 1980s when the importance of heat transfer mechanisms [3, 4] in image interpretation was understood. Now, infrared thermography is a mature technique and is becoming ever more attractive in an ever more increasing number of application fields. This has also led to a proliferation of infrared devices, which differ in weight, dimensions, shape, performance, and costs, to fulfil desires of a multitude of users in a vast variety of applications [5]. In fact, an infrared imaging system can be now tailored for specific requirements and it can be advantageously exploited for process control and maintenance planning without production stops and with consequent money saving. Of course, complete exploitation of infrared thermography requires understanding of basic theory and application of standard procedures. 

Of relevant interest is the application of an infrared imaging device in architecture and civil engineering after Building Regulations for Conservation of Fuel and Energy (2007). However, infrared thermography can be used also to discover defects in buildings envelope, to monitor reinforcing steel in concrete, to detect moisture inside building walls, and so forth [6–8]. 

It is known that masonry structures deteriorate over time mainly due to natural forces of decay, due to thermal stresses, and due to water infiltration; the main degradation effects include variations in concrete compaction and voiding, spalling or microcracking in masonry, and reinforcement deterioration and this may be of great concern if the structure belongs to the cultural heritage. Indeed, IRT represents a valuable tool for nondestructive evaluation of architectonic structures and artworks because it is capable of giving indication about most of the degradation sources of artworks and buildings of both historical interest and civil use. In particular, by choosing the most adequate thermographic technique, it is possible to monitor the conservation state of artworks in time and to detect the presence of many types of defects (e.g., voids, cracks, disbonding, etc.) in different types of materials [9, 10]. It is possible to inspect either a large surface, such as the facade of a palace, or a very small surface of only few square millimetres. The main advantages of infrared thermography when dealing with precious artworks may be summarized in three words: noncontact, noninvasive, and two dimensional. 

Long-term conservation of artworks involves periodic inspection to evaluate existing conditions, to discover deficiencies at an incipient stage, and to plan restoration before catastrophic failure occurs. In this context, infrared thermography (IRT), as a remote imaging system, represents a powerful tool to be used for quick periodic inspection. The images can be stored in a digital format and a history of the material degradation can be easily examined and visualized as well as compared to a previous situation by retrieval of archived images. It is known that IRT has some limitations when dealing with deep and low thermal resistance defects, but it has proved to still be useful in conjunction with high-depth techniques [11–13].

This work would be an overview of some of the applications of infrared thermography to the architectural field performed at the Aerospace Engineering Section of the Department of Industrial Engineering, University of Naples Federico II, to which the author belongs. The results shown herein come from laboratory tests as well as in situ inspection of civil buildings and of important artworks such as the mosaic of the Battle of Issus in the Archaeological Museum of Naples and frescoes in the Villa Imperiale in Pompeii. 

## 2. Instrumentation and Test Procedure

Tests were carried out by using pulse and lock-in techniques and different cameras which include both cooled and uncooled detectors. A brief description of the two techniques and infrared cameras is given as follows.

### 2.1. Pulse Thermography

Pulse thermography (PT) consists simply in the stimulation of the object (under evaluation) and monitoring of its surface temperature variation during the transient heating, or cooling, phase. Heating is generally performed by lamps, flash lamps, scanning lasers, or hot air jets. Cooling can be practically attained by cold air jets. Of course, air jets (hot or cold) can be used only on a massive surface since jet impingement may damage delicate objects, for example, artworks. 

For the case of slabs, analysis with PT can be performed in two different modes: transmission and reflection. In the transmission mode, the infrared camera views the rear face, that is, the face opposite to the heating/cooling source. However, since the opposite side is not always accessible and/or available, the reflection mode, for which both heating (cooling) source and camera are positioned on the same side, is mainly applied. 

The thermal energy propagates by diffusion under the surface while the infrared camera monitors the temperature variation over the viewed surface. Obviously, for a uniformly heated surface, the temperature distribution is uniform in case of a homogeneous material. The presence of a defect at a certain depth interferes with the heat flow causing local surface temperature variations. The visibility of a defect can be evaluated by the following parameter *D*_*T*_ [14]:

(1) DT=|ΔT||ΔTs|=|Ts−Td||Ts−Tr|,

where *T*_*d*_ is the temperature over a defective zone, *T*_*s*_ is the temperature in a sound zone, and *T*_*r*_ is a reference temperature. More specifically, *T*_*r*_ is the temperature of the sound material before starting transient heating, or cooling, *T*_*r*_ = *T*_*s* (*t* = 0)_ (i.e., the temperature of the first thermal image taken at *t* = 0 s in the time sequence). Indeed, the quantity *T*_*s*_ − *T*_*r*_ = Δ*T*_*s*_ plays an important role because it indicates the optimal temperature variation to which the material has to be subjected for good defect visibility. 

The defect detection is limited by the signal-to-noise ratio (SNR) [15] or by the noise equivalent temperature difference (NETD) of the infrared detector. In addition, the surface finish is of great importance since variations in surface roughness, cleanliness, uniformity of paint, and other surface conditions can cause variations in the emissivity coefficient and affect the temperature measurement. These drawbacks are overcome using lock-in thermography.

### 2.2. Lock-In Thermography

In this work, lock-in thermography is performed with halogen lamps and is simply referred to as LT. The energy, generated by halogen lamps, is delivered to the object surface in the form of periodic thermal waves. The thermographic system is coherently coupled to the thermal wave source which is operated in such a way that a sinusoidal temperature modulation results. This modulation is obtained from a nonlinear electrical signal produced by the lock-in module which also allows for frequency variation. The thermal wave propagates inside the material and gets reflected when it reaches parts where the heat propagation parameters change (inhomogeneities). 

The reflected wave interferes with the surface wave, producing an oscillating interference pattern, which can be measured in terms of amplitude, or phase angle, that, respectively, produces amplitude, or phase, images. The depth range for the amplitude image is given by *μ* which is calculated from the thermal diffusivity *α* and the wave frequency *f* = *ω*/2*π* as follows:

(2) $$\begin{array}{c} \mu =\sqrt{\frac{\alpha }{\pi f}}. \end{array}$$

The maximum depth *p*, which can be reached for the phase image, corresponds to 1.8 *μ* [16–19]. The material thickness, which can be inspected, depends on the wave period (the longer the period, the deeper the penetration) and on the material properties (e.g., thermal diffusivity).

### 2.3. Infrared Cameras

Different infrared cameras by FLIR systems are used which include either high performance cooled detector cameras, which are suitable for the research field, or handheld cameras that are more appropriate for in situ inspections. All the handheld cameras are equipped with microbolometer detectors working in the long wave infrared band generally from 8 up to 12 microns. They differentiate for thermal sensitivity and mainly for spatial resolution. In particular, the B4 and B360 have both 320 × 240 pixels, but the B360 offers the advantage of a more ergonomic configuration with the possibility to rotate the lens and take images also in presence of problematical optical access. Of the same ergonomic configuration but of better resolution is the T640 which has 640 × 480 pixels; the same number of pixels both P640 and P660 also have, the latter being an upgrade of the P640 with obviously a better performance. As a general characteristic, for all the new generation handheld cameras, images are stored on flash memory cards. 

The cooled detector cameras used by the author for architectural applications include four cameras. Two cameras, equipped with a single MCT detector working in the 8–12 microns and cooled with liquid nitrogen, are the Agema 880 of 140 × 140 pixels and digitalization at 8 bits and the Agema 900 of 272 × 136 pixels and digitalization at 12 bits. Two cameras, including FPA QWIP second generation sensors working in the 8-9 microns with Stirling cooler, are the SC3000 of 320 × 240 pixels and the SC6000 of 640 × 512 pixels (FLIR systems). Herein, only some peculiar results obtained by either uncooled or cooled detector cameras are shown as examples.

## 3. Tests and Data Analysis 

Tests were carried out partly in laboratory to assess testing procedures and data analysis and partly in situ including either civil habitations or archaeological sites as well as works of art. 

### 3.1. Laboratory Tests

Laboratory tests are carried out by considering two types of specimens which simulate one- and two-layer structures, with defects of different geometry and nature, located at different depths [20]. 

**Figure 1:**
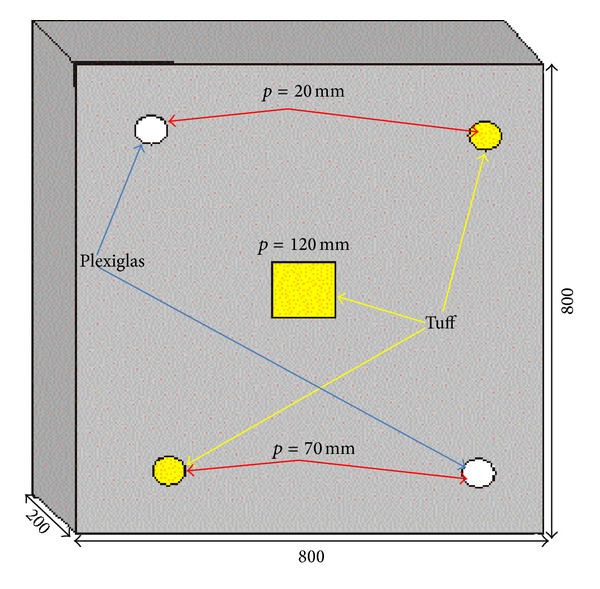
Sketch of one-layer specimen.

The one-layer specimen, of square section 800 × 800 mm^2^ and of thickness 200 mm, is made of concrete (a mixture of cement, sand, and water) with buried defects (air-filled Plexiglas boxes and pieces of tuff) at depth from *p* = 20 mm up to 120 mm (Figure 1). More specifically, the four circular inserts are 70 mm in diameter, while the central square-shaped insert has a side of 100 mm; the thickness of all is 30 mm.

**Figure 2:**
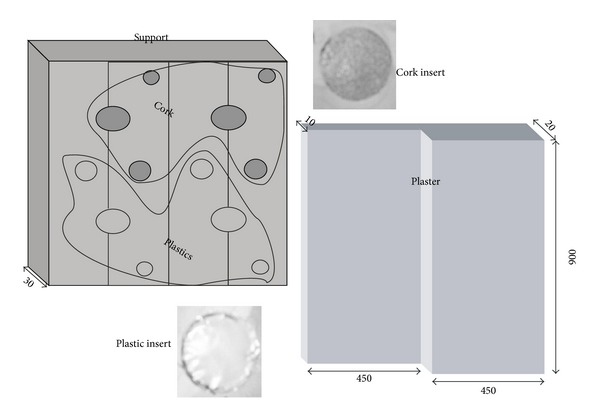
Sketch of two-layer specimen.

The other type, of square section 900 × 900 mm^2^, is composed of a plaster layer (one half is 10 mm and the other half is 20 mm thick) over a support of brick, marble, or tuff with cork disks and air-filled plastic bags (in total 12) between the plaster and the support to simulate detachments (Figure 2). The inserts are 1 mm thick and of diameter ranging between 40 mm and 100 mm.

**Figure 3:**
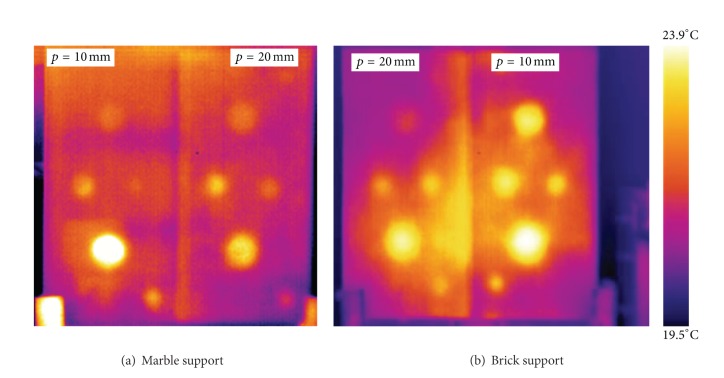
Thermal images of two two-layer specimens.

The results herein reported are obtained with the cooled FPA detector SC3000 and by using the PT technique. Two thermal images of two-layer specimens are shown in Figure 3 for the support made of marble (Figure 3(a)) and of brick (Figure 3(b)). As can be seen, all defects are visible, but the higher contrast is caused by the larger air-filled Plexiglas boxes. It is worth noting that the images in Figure 3 are taken at a certain time instant, during the cooling phase, for which all defects are visible; in fact, shallow defects (at depth *p* = 10 mm) appear first, but they tend to vanish later when the deeper (*p* = 20 mm) ones become visible. Indeed, the visibility of a defect depends strongly on its thermal resistance which, in turn, depends on both geometrical (i.e., diameter, thickness, and depth) and thermal characteristics (i.e., conductivity, diffusivity, and effusivity); an important role is of course played by the characteristics (resolution) of the used instrumentation [20].

**Figure 4:**
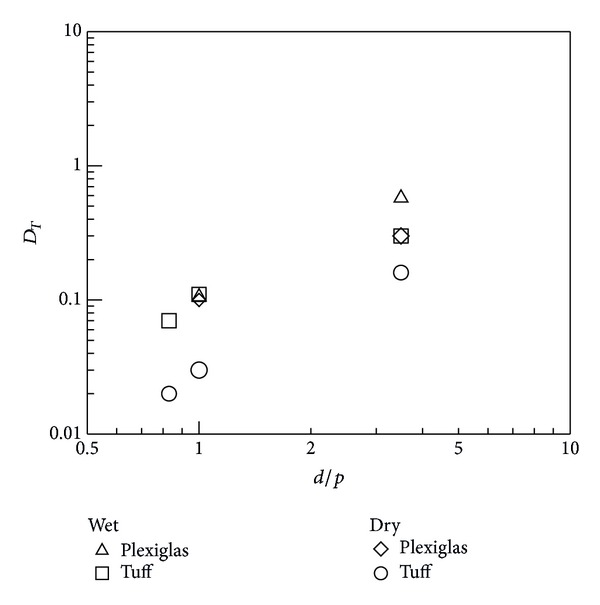
*D*_*T*_ against *d*/*p* for the one-layer specimen.

The contrast *D*_*T*_ (see (1)) is plotted against the defects diameter over depth ratio *d*/*p* in Figure 4 for the one-layer specimen considering both completely dry and partially wet concrete (tests are carried out two times: three months after fabrication and six months later). As can be seen, the tuff inserts display higher contrast for wet conditions; on the other hand, tuff, in presence of water, gets soaked, modifying its thermal properties with respect to the surrounding masonry structure. Plexiglas instead, being waterproof, has the same thermal contrast independent of the moisture content of the hosting material.

### 3.2. Inspection In Situ

Infrared thermography was widely used for in situ inspection of architectonic structures and works of art [9, 11–14]. Only a few examples are reported herein.

#### 3.2.1. Inspection of Civil Edifices

**Figure 5:**
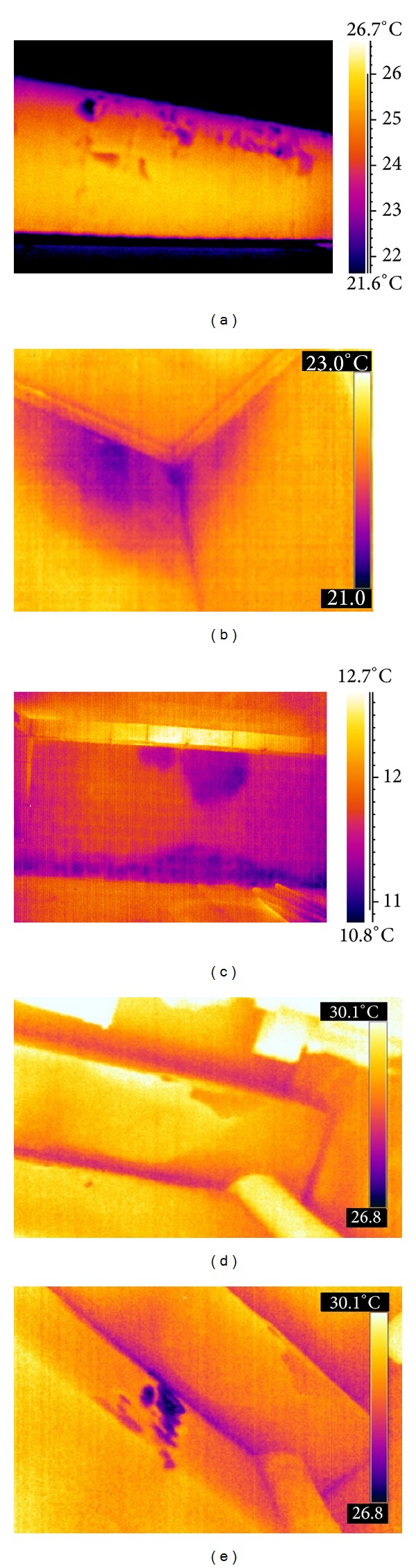
Examples of water infiltration. (a) Underneath surface of a balcony. (b) Wall corner inside a house. (c) Wall surface inside a gym. (d) Garage roof. (e) Garage roof during forced infiltration.

Some thermal images accounting for infiltration of water and presence of humidity are shown in Figure 5, for outside (Figure 5(a)) and inside (Figures 5(b)–5(e)). All images are taken with handheld cameras, more specifically, with the B4 (the images shown in Figures 5(a), 5(d), and 5(e)), with the B360 (the image shown in Figure 5(b)), and with the P640 (the image shown in Figure 5(c)). Figure 5(a) shows the underneath surface of a balcony; the dark stains indicate degradation of concrete because of infiltration of water most probably due to poor tightness of the upside surface. Figure 5(b) represents the corner between walls of a house in which a humidity stain is clearly evident. Humidity stains are also present on the wall of the public edifice, which is a sports centre, as shown in Figure 5(c). In the latter, it is possible to see humidity stains developing on the top due to not only poor tightness of the external wall envelope, but also humidity raising from the bottom through capillary suction.  Figure 5(d) shows a portion of a garage roof in which there is water infiltration; Figure 5(e) represents the same scene as Figure 5(d) but after forced infiltration of water with the aim of locating the entrance pathway. From a comparison between images, it is possible to distinguish infiltration in progress, or better the water pouring out as in Figure 5(e), from the effects of infiltration like the damp patches in Figures 5(a)–5(c). 

Water infiltration is a complex task to deal with since it is very difficult, or impossible, to precisely locate the entry point without disruption of the roof, or pavement; this is because from entrance and exit, often, there may be a tortuous pathway. In addition, often, water infiltration remains imperceptible to naked eyes for a long time, becoming noticeable when the unpleasant damp patches appear; this happens because of the diffusion aptitude of water. An infrared imaging device may help solve the problem through a suitable procedure involving forced water entrance; this is the approach pursued to discover water infiltration in the roof of the garage (Figure 5(e)). 

**Figure 6:**
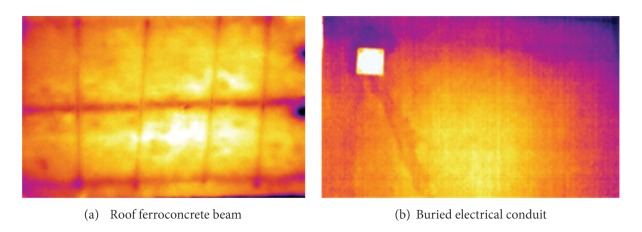
Examples of thermal images in presence of buried structures.

An example of buried structures is given in Figure 6. The first image (Figure 6(a)) is taken at a distance of 3 m with pulse thermography and with the SC3000 camera. It refers to a roof ferroconcrete beam in a warehouse; the thicker dark horizontal lines represent the buried steel rods, while the vertical milder lines indicate brackets. The beam under study is 40 cm wide and about 15 m long and it is tapered along the third dimension. It is interesting to see that infrared thermography offers the possibility to ascertain the presence of steel bars inside ferroconcrete beams and to evaluate their diameter. This, of course, is of paramount importance since it prevents from destructive tests and is time and money saving. The second image, taken with the B360 camera (Figure 6(b)), displays irregular dark bands that indicate the presence of buried duct cables there. Often, as in this case, conduits for electrical cables do not follow straight directions, while finding their exact location is vital to avoid troubles when drilling a hole in the wall. 

#### 3.2.2. Application to the Cultural Heritage

The increasing sensitivity towards the conservation of cultural properties is looking at infrared thermography as an excellent aid [10]. The main causes of degradation of art treasures lie in exposure to adverse environmental conditions including thermal and mechanical stresses and variation of humidity which give rise to microcracks, disbonding, and formation of mould. It is of utmost importance to detect defects at an incipient stage and to understand modifications induced by variation of environmental parameters to plan the most adequate program of prevention from decay of artworks. Infrared thermography has proved its capability to help find detached tiles in mosaics and degradation of plaster and frescoes [9], to measure thermal properties like diffusivity and effusivity of materials for the solution of the inverse problem [10], and to monitor microclimatic conditions [21] which often represent the major hazard issue for the conservation of precious works of art.

**Figure 7:**
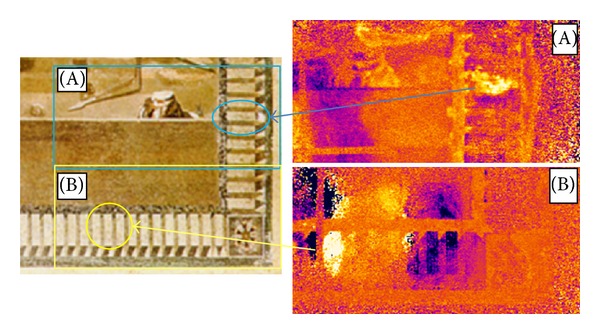
A part of the Battle of Issus mosaic (Archaeological Museum in Naples).

**Figure 8:**
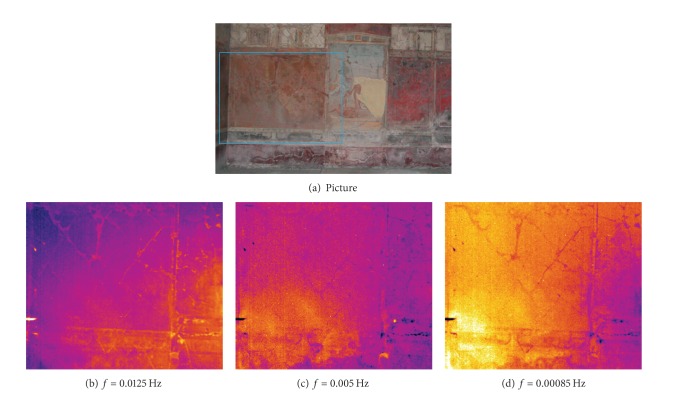
Phase images ((b)–(d)) on the wall (a) in the oecus room in Villa Imperiale (Pompeii).

Herein, some phase images collected during campaigns of tests in situ in the Archaeological Museum of Naples and in the archaeological site of Pompeii are shown. Such images are taken with cooled detector cameras (Agema 900 and SC3000) equipped with the IR lock-in option. In particular, phase images reported in Figure 7 are taken with the Agema 900 camera, while phase images reported in Figure 8 are taken with the SC3000 camera.

Figure 7 shows a picture of a portion of the famous mosaic of the Battle of Issus (Archaeological Museum in Naples) with two phase images. This is a masterpiece of inestimable value, which was created with the opus vermiculatum technique; tiles of miniature size (about 20 tiles in a cm^2^) were glued with rosin, which is sensitive to temperature rise. Then, the inspection of such artwork poses serious problems in terms of its safeguard. As a general comment, the PT technique is not suitable since it is affected by nonuniform emissivity distribution which is a major problem in the evaluation of mosaics, because, to obtain the desired chromatic effects, tiles of different materials, colours, and brightness are generally used and this causes local variation of emissivity. Thus, the evaluation is made with the LT technique and with special care to avoid undesired temperature rise; the lamp is positioned far enough from the mosaic surface. The variation of the phase angle with varying heating frequencies provides information useful to discriminate between tiles made of different materials, to locate disbonded tiles and detachments in the plaster support underneath the tiles, and to discover restored plaster in areas without tiles. In particular, the two phase images taken both at *f* = 0.019 Hz, which refer the one on the right top side to the surface encircled by rectangle A and the other one on the right bottom side to the surface encircled by rectangle B, supply relevant details. More specifically, the variation of colour (variation of phase angle) indicates variation of material compaction with presence of detachments in the encircled zones. 

Figure 8(a) shows a picture of a frescoed wall of the oecus room of Villa Imperiale in Pompeii, while Figures 8(b)–8(d) show phase images taken at decreasing frequency of the surface inscribed inside the rectangle in Figure 8(a). It is possible to see a crack network which is present on the surface (Figure 8(b)) and propagates underneath in the support (Figure 8(d)). The lighter colour in Figure 8(c) indicates a general degradation of the support underneath the frescoes; such a degradation is more important on the left corner as the lighter colour intensity shows (Figure 8(d)). From a comparison between the three images, it is also possible to gain information about the junction between the central panel and the left wall. In particular, the phase image in Figure 8(c), which was taken at the heating frequency of 0.005 Hz (depth *p* about 1.2 cm), shows, in agreement with GPR data [13], a consolidation structure across the junction of the left wall part to the central panel. 

## 4. Conclusions

Some of the results obtained while applying infrared thermography to the inspection of architectonic structures and works of art have been illustrated with also a brief introduction to the basic procedures. As main findings, the applied techniques allow for the detection of hidden structures, as well as for the identification of areas damaged by in-depth ingression of moisture and/or by disaggregation of the constituent materials. The attention herein has been mainly devoted to the conservation conditions of masonry structures, while an infrared camera can be advantageously exploited also for the control of microclimatic conditions.

Indeed, infrared thermography, as a remote imaging device, represents a powerful tool to be used for quick periodic inspection of both civil habitations and historic buildings to discover defects in buildings envelope, to monitor reinforcing steel in concrete, to detect moisture inside building walls, to check indoor microclimate, and so forth. All this information is useful to deal with conservation measures and to improve microclimatic conditions for the comfort of human beings and for the safeguard of the works of art.
